# Chronic Kidney Disease and Gut Microbiota: What Is Their Connection in Early Life?

**DOI:** 10.3390/ijms23073954

**Published:** 2022-04-02

**Authors:** Chien-Ning Hsu, You-Lin Tain

**Affiliations:** 1 Department of Pharmacy, Kaohsiung Chang Gung Memorial Hospital, Kaohsiung 833, Taiwan; cnhsu@cgmh.org.tw; 2 School of Pharmacy, Kaohsiung Medical University, Kaohsiung 807, Taiwan; 3 Department of Pediatrics, Kaohsiung Chang Gung Memorial Hospital and Chang Gung University College of Medicine, Kaohsiung 833, Taiwan; 4 Institute for Translational Research in Biomedicine, Kaohsiung Chang Gung Memorial Hospital and Chang Gung University College of Medicine, Kaohsiung 833, Taiwan

**Keywords:** chronic kidney disease, hypertension, children, short-chain fatty acids, developmental origins of health and disease (DOHaD), gut microbiota, probiotics, prebiotics, trimethylamine-N-oxide

## Abstract

The gut–kidney interaction implicating chronic kidney disease (CKD) has been the focus of increasing interest in recent years. Gut microbiota-targeted therapies could prevent CKD and its comorbidities. Considering that CKD can originate in early life, its treatment and prevention should start in childhood or even earlier in fetal life. Therefore, a better understanding of how the early-life gut microbiome impacts CKD in later life and how to develop ideal early interventions are unmet needs to reduce CKD. The purpose of the current review is to summarize (1) the current evidence on the gut microbiota dysbiosis implicated in pediatric CKD; (2) current knowledge supporting the impact of the gut–kidney axis in CKD, including inflammation, immune response, alterations of microbiota compositions, short-chain fatty acids, and uremic toxins; and (3) an overview of the studies documenting early gut microbiota-targeted interventions in animal models of CKD of developmental origins. Treatment options include prebiotics, probiotics, postbiotics, etc. To accelerate the transition of gut microbiota-based therapies for early prevention of CKD, an extended comprehension of gut microbiota dysbiosis implicated in renal programming is needed, as well as a greater focus on pediatric CKD for further clinical translation.

## 1. Introduction

Up to 10% of the population worldwide is affected by chronic kidney disease (CKD) [1]. CKD can be attributed to different negative conditions in early life [2,3,4], and therefore, World Kidney Day 2016 made efforts to keep the public informed of the need to focus on kidney disease in childhood and the antecedents of adult kidney disease [5]. During development, the fetal kidney is susceptible to a suboptimal in utero environment, resulting in alterations in function and structure by so-called renal programming [6]. The phenomenon of adverse conditions during organ development resulting in adult disease in later life is now termed “developmental origins of health and disease” (DOHaD) [7]. Conversely, adverse fetal programming could be reprogramming before clinical onset of the disease by early therapeutic intervention [8]. Accordingly, a shift of focus from treatment of established CKD towards the prevention of kidney disease in the earliest stage is highly needed.

Although various organ systems can be programmed in response to in utero suboptimal conditions, renal programming is considered key in the development of CKD and its comorbidities [6,9]. Renal programming is likely to constitute a first hit to the kidney, which makes the kidney more vulnerable to postnatal insults (i.e., second hit) to develop CKD in later life. Up to now, researchers have proposed some mechanisms associated with renal programming. These mechanisms, such as dysregulated nutrient-sensing signals [9], oxidative stress [10], nitric oxide (NO) signaling [11], aberrant activation of the renin–angiotensin system (RAS) [12], and gut microbiota dysbiosis [13,14], have been contributing to CKD in later life [2,3,4,6,8,9].

Due to the low antioxidant capacity of embryos [15], the developing kidney is extremely vulnerable to oxidant stress injury. As reviewed elsewhere [16], a number of animal models support that NO/reactive oxygen species imbalance-induced oxidative stress is involved in renal programming. On the other hand, increasing evidence suggests antioxidants can be used as reprogramming strategies to prevent kidney disease and hypertension of developmental origins [17]. In the developing kidney, the RAS components are highly expressed and play a key role in mediating proper physiological function and renal morphology [18]. A transient biphasic response with downregulation of the classical RAS axis in the neonatal stage becomes normalized with age [19,20]. Data from renal programming models reported that various early-life insults can disturb this normalization in adults, and consequently, the classical RAS axis is inappropriately activated, leading to the development of kidney disease in adulthood [6,19,20]. Conversely, emerging evidence supports that early RAS-based interventions could reverse programming processes to prevent kidney disease of developmental origins [12]. Additionally, nutrient-sensing signals play an essential role in normal renal physiology and the pathogenesis of kidney disease [21]. Early-life nutritional insults can impair nutrient-sensing signals that affect fetal development and, consequently, program chronic disease in later life [22]. Dysregulated nutrient-sensing signals, such as AMP-activated protein kinase (AMPK) and peroxisome proliferator-activated receptors (PPARs), have been linked to renal programming and the risks for developing kidney disease in later life [23,24]. Despite the fact that the complete mechanisms are still inconclusive, there seem to be interrelated aspects among them. Since detailed reviews of each mechanism are beyond the scope of this paper, readers are referred elsewhere [8,9,10,11,12,13,14].

Recent studies have focused on the impact of the gut microbiome in CKD and its associated complications [14]. Microbial metabolites can act as signaling compounds via systemic circulation [14]. Currently, there are some proposed mechanisms linking dysbiotic gut microbiota to CKD and related complications, such as alterations of the gut microbiome, dysregulation of short-chain fatty acids (SCFA) and their receptors, activation of aryl hydrocarbon receptor (AHR), increases of trimethylamine-N-oxide (TMAO), and microbiota-derived uremic toxins [14,25,26,27,28,29]. Maternal insults have been shown to change gut microbiome balance, leading to an increased risk of adult diseases [29]. Nevertheless, relatively little is known about whether and how diverse prenatal insults could influence gut microbiota, leading to CKD and its comorbidities in adult offspring.

This scoping review followed the Preferred Reporting Items for Systematic Reviews and Meta-Analyses extension for Scoping Reviews (PRISMA-ScR) to identify and examine the evidence around the impact of gut microbiota behind the programming of kidney disease evidence documenting prevention of CKD and its related complications by early-life gut microbiota-targeted therapy [30]. Our search strategy was designed to retrieve literature relating to DOHaD, gut microbiota, and pediatric kidney disease from PubMed/MEDLINE databases. We used the following search terms: “chronic kidney disease”, “developmental programming”, “DOHaD”, “reprogramming”, “gut microbiota”, “probiotics”, “prebiotics”, “synbiotics”, “postbiotics”, “mother”, “pregnancy”, “gestation”, “offspring”, “progeny”, “uremic toxin”, “nephrogenesis”, “nephron number”, “kidney”, “aryl hydrocarbon receptor”, and “hypertension”. Additional studies were then selected and assessed based on fitting references in eligible papers. The last search was conducted on 25 January 2022.

## 2. Human Evidence for Developmental Programming of CKD

The development of the human kidney starts at week 3 and ends at week 36 of gestation [31]. Hence, term neonates are born with a full complement of nephrons. In each kidney, the average number of nephrons, the basic unit of a kidney, is approximately 1 million with 10-fold interindividual differences [32]. Adverse in utero events could interfere with nephrogenesis, resulting in a reduction of nephron numbers and a wide range of congenital anomalies of the kidney and urinary tract (CAKUT) [33]. Reduced nephron number causes glomerular hyperfiltration and compensatory glomerular hypertrophy, consequently initiating a vicious cycle of further nephron loss [34]. Accordingly, reduced nephron number could act as a first trigger to increase the offspring’s vulnerability to CKD throughout their later life.

Strong support for the developmental programming of CKD came from a number of epidemiological studies. Premature birth and low birth weight (LBW) are significant risk factors for CKD in later life [35,36,37,38]. A meta-analysis study recruiting more than 2 million babies revealed that LBW babies were 70% more likely to develop CKD in later life than those with normal birth weight [36]. In addition to premature birth and LBW, a case–control study of 1.6 million infants revealed that maternal gestational diabetes, maternal thalassemia/hemochromatosis, male gender, polyhydramnios or oligohydramnios, and first pregnancy are also risk factors for CAKUT [37]. Another case–control study recruiting 2000 CKD children acknowledged several early-life risk factors, such as LBW, prematurity, gestational diabetes, and maternal obesity, showed an increased risk of CKD in adult life [38]. As we reviewed elsewhere, a number of environmental risk factors are related to the developmental programming of CKD, such as maternal illness, nutritional imbalance, environmental chemicals, medication use, substance abuse, infection, and exogenous stress [4]. For example, maternal obesity and diabetes are correlated with an increased risk of kidney disease in adulthood [39,40]. Additionally, deficiencies in maternal total energy [41], folate [42], and vitamin A [43] during pregnancy were associated with detrimental influence on kidney structure and function. Epidemiological studies also showed that maternal exposure to polycyclic aromatic hydrocarbon, per- and polyfluoroalkyl substances, and polycyclic aromatic hydrocarbon, as well as air pollution associated with premature birth and LBW [44,45,46,47], are both risk factors for low nephron number. Moreover, a number of drugs administrated to pregnant women have been known to affect kidney development, resulting in CAKUT [48]. These medications include angiotensin converting enzyme inhibitor, angiotensin receptor blockers, aminoglycosides, cyclosporine A, dexamethasone, furosemide, anti-epileptic drugs, cyclophosphamide, etc. [48].

Although the risk of CKD has been evaluated in plenty of human studies, interventions required to prove causation and to elucidate underlying molecular mechanisms remain unknown. Most of our knowledge regarding the critical window of vulnerability for insults, the types of insults driving renal programming, potential core mechanisms behind renal programming, and therapeutic strategy arise out of studies in animal models.

## 3. Gut Microbiota and Kidney Disease

Trillions of microbes living in the gut—the gut microbiota—have coexisted with humans in a state of mutually beneficial cohabitation. A diversity of environmental factors can induce gut microbial imbalance (i.e., dysbiosis), which in turn can affect human health and disease [49]. Although the role of gut microbiota in adulthood advanced CKD has been extensively reviewed elsewhere [14,25,50,51], less attention has been given to investigate its impact in early stages of kidney disease. Therefore, this section mainly discusses evidence supporting the role of early-life gut microbiota in humans, with an emphasis on pediatric CKD.

### 3.1. Early-Life Gut Microbiome

Although microbes colonize the neonatal gut immediately following birth [52], microbial colonization continues to develop and vary in species abundance until a typical adult-like gut microbiota is established at the age of 2–3 years [53]. A variety of maternal factors and early-life events determine the establishment of the gut microbiome, such as gestational age, type of delivery, maternal conditions, formula feeding, antibiotic exposure, and ecological factors [52,53,54,55].

During pregnancy and lactation, the mother gut microbiota can influence offspring gut microbial structure and composition, which highlights the importance of maternal factors in the establishment of early-life gut microbiome [55]. Several risk factors related to CKD of developmental origins have also been linked to alterations of gut microbiota, such as gestational diabetes [56], maternal obesity [57], prematurity [58], LBW [59], and maternal malnutrition [60]. Additionally, the establishment of the microbiome is highly interconnected with development of the immune system, and CKD has strong immune and inflammatory etiologies [61].

Moreover, several environmental chemicals that pregnant mothers are likely to be exposed to are associated with developmental origins of kidney disease [62]. Among them, exposure to heavy metals, polycyclic aromatic hydrocarbons, and dioxins also affect the gut microbiome, accompanied with the development of adult diseases [63]. All of these studies suggest that the early-life microbial alterations after the CKD-related adverse insults may be involved in the development of kidney disease in later life.

### 3.2. The Gut–Kidney Axis

**Figure 1:**
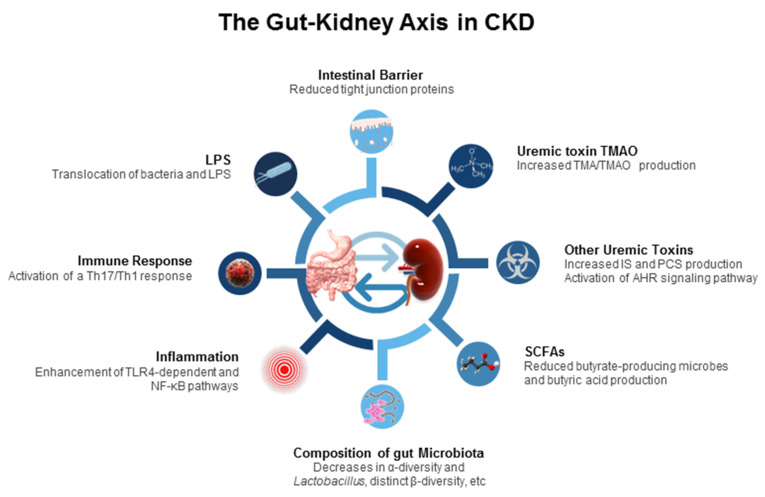
Schematic diagram summarizing the proposed mechanisms related to the gut–kidney axis involved in the pathogenesis of chronic kidney disease and its comorbidities. LPS = lipopolysaccharide; Th17 = T-helper 17 cell; Th1 = T-helper 1 cell; TLR4 = toll-like receptor 4; NF-κB = nuclear factor kappa B; SCFA = short-chain fatty acid; IS = indoxyl sulfate; PCS = p-cresyl sulfate; AHR = aryl hydrocarbon receptor; TMA = trimethylamine; TMAO = trimethylamine-N-oxide.

The pathogenic interconnection between the gut microbiome and kidney diseases is termed the gut–kidney axis [14], which is implicated in CKD and its comorbidities. A paucity of data exists regarding how the gut–kidney axis functions in the pediatric population with CKD and what the impact of the gut microbiota is in this process. However, a great deal of work on the impact of the gut–kidney axis in established CKD has been conducted, including gut barrier dysfunction, inflammation, immune response, alterations of microbiota compositions, dysregulated SCFAs and their receptors, uremic toxins, etc. (Figure 1). Each of them are discussed.

First, CKD can impair the intestinal barrier by disrupting the epithelial tight junction in a 5/6 nephrectomy rat model [64]. An apparent reduction of the tight junction proteins was reported in the gut mucosa of CKD animals, possibly attributed to uremic toxins [44]. As a result, an increased intestinal permeability and translocation of lipopolysaccharide (LPS) and bacteria across the intestinal barrier were reported. In CKD rats, gut bacteria could activate a T-helper 17 (Th17)/Th1 T-cell response and increase the production of inflammatory cytokines, and LPS could initiate innate immune cells through nuclear factor kappa B (NF-κB) and toll-like receptor 4 (TLR4) pathways, all triggering inflammation and immune response [65].

Second, changes in the composition of the gut microbiota are relevant to CKD. Uremia profoundly alters 190 and 175 bacterial operational taxonomic units (OTUs) of the gut microbiome in CKD humans [66] and rats [67], respectively. Specifically, the presence of aerobic bacteria such as those belonging to the phyla *Firmicutes*, *Actinobacteria*, and *Proteobacteria* in higher numbers, but fewer anaerobic bacteria, such as *Sutterellaceae*, *Bacteroidaceae*, and *Lactobacillaceae*, were observed in end stage kidney disease (ESKD) [45,46,47]. Notably, most research has consistently reported that animals and adult patients with CKD had low abundance of genus *Lactobacillus*, whereas the proportion of family *Enterobacteriaceae* were increased [14,66,67,68,69]. A systemic review recruiting 25 studies with 1436 CKD patients revealed that the α-diversity was decreased, and β-diversity of gut microbiota was significantly more distinct in ESKD patients than in healthy controls [69].

Third, the gut microbiota produces diverse metabolites, which are involved in multiple physiological processes, such as immunity and host energy metabolism [14]. Following dietary exposures to certain nutrients, particular microbiota-derived metabolites could be altered in ESKD patients [70]. Carbohydrates are fermented to generate SCFAs which signal the host to increase energy expenditure, enhance G protein-coupled receptor (GPCR) signaling, and act as an inhibitor for histone deacetylase (HDAC) [70,71,72]. SCFAs are made up of one to six carbon atoms (C1–C6), mainly consisting of acetic acid (C2), propionic acid (C3), and butyric acid (C4) [71]. In adult CKD patients, butyrate-producing microbes and butyric acid production reduced with disease severity [73].

Indoxyl sulfate (IS) and p-cresyl sulfate (PCS), both end-products of protein fermentation, and TMAO, an end-product of microbial carnitine/choline metabolism, are well-known microbiota-derived uremic toxins. Urinary excretion of several microbial tryptophan metabolites such as IS and PCS is decreased in patients with CKD. These tryptophan metabolites mainly from the indole metabolic pathway are accumulated as uremic toxins, which are ligands for AHR [74]. Activation of AHR is able to trigger inflammation, induce oxidative stress, and modulate the Th17 axis, contributing to CKD progression in vivo and in vitro [75,76]. The level of another uremic toxin, TMAO, is high in patients with ESKD and associated with increased risk of cardiovascular disease [77,78]. TMAO generation results from the fermentation by the gut microbiota of dietary carnitine/choline, which is converted to trimethylamine (TMA) and transformed into TMAO by flavin-containing monooxygenase (FMO) in the liver. Conversely, selective targeting of gut-microbiota-dependent TMAO generation has been reported to protect CKD progression in a murine model of CKD [79]. Although the uses of prebiotics, probiotics, postbiotics, and synbiotics have shown potential positive effects against uremic toxin generation, their evidence is still limited for the treatment and prevention of human CKD [80,81,82].

Together, the interaction between gut microbiota and CKD is bidirectional: CKD may affect the structure of the gut microbiota and contribute to gut dysbiosis, while dysbiosis in CKD patients may increase uremic toxin levels that in turn contribute to CKD progression. Considering the gut is a potential cause of CKD-related complications, gut microbiota-targeted therapeutic strategies in CKD will have a considerable impact on CKD management.

### 3.3. Gut Microbiota in Pediatric CKD

Table 1 summarizes the alterations of gut microbiota and its related metabolites in pediatric kidney disease, as reported in the literature [83,84,85,86,87,88,89]. The study of the gut microbiome in children with kidney disease mainly focused on three types of dysbiosis: loss of diversity, shifts in keystone taxa, and alterations of microbial metabolites.

The pediatric gut microbiome in a uremic milieu has been evaluated in a small group of ESKD children who underwent hemodialysis (HD, *n* = 8), peritoneal dialysis (PD, *n* = 8), or kidney transplant (*n* = 10) [83]. Alpha diversity was decreased in children undergoing PD or transplant. ESKD children undergoing HD had increased abundance of phylum *Bacteroidetes*. Children on PD had an increase in the abundance of phyla *Firmicutes* and *Actinobacteria* but a decrease in abundance of family *Enterobacteriaceae*. Additionally, children on HD or PD had increased plasma levels of microbiota-derived uremic toxins, IS, and PCS [83]. A similar pattern of gut dysbiosis was reported in adult patients with ESKD [69,70].

In another small group of children (*n* = 12) with idiopathic nephrotic syndrome (INS), butyric acid level in the feces was decreased in relapsing INS children coinciding with decreased abundance of butyrate-producing bacteria belonging to *Clostridium* clusters IV and XIVa [84]. These microbes included *Clostridium orbiscindens*, *Faecalibacterium prausnitzii*, *Eubacterium hallii*, *E. ramulus*, *E. rectale*, *E. ventriosum*, *Roseburia intestinalis*, *Eubacterium* spp., and *Butyrivibrio* spp.

One study recruiting 60 children diagnosed with CKD stage 1 and 26 stage 2–3 children showed that urinary levels of TMAO and dimethylamine (DMA, a metabolite of TMAO) were lower in children with CKD stages 2–3 than CKD stage 1 [85]. Additionally, the proportion of genus *Prevotella* was decreased in CKD children with blood pressure (BP) abnormalities.

In 78 children and adolescents with CKD stage 1–4 and a median age of 11.2 years, BP determined using 24 h ambulatory blood pressure monitoring (ABPM) was defined out of range, and BP was related to increased plasma levels of propionic acid and butyric acid [86]. Additionally, the abundance of phylum *Verrucomicrobia*, genus *Akkermansia*, and species *Bifidobacterium bifidum* were higher in CKD children with CAKUT compared to those with non-CAKUT. 

In another study from our group, we recruited 115 children and adolescents with CKD stages 1–4 [88]. We found plasma levels of DMA, trimethylamine (TMA), and TMAO higher in children with CKD stage 2–4 vs. CKD stage 1. These data are consistent with previous studies in CKD adults [90,91], showing that TMAO is increased in CKD and that there is a negative association between circulating TMAO level and renal function. We also observed that phylum *Cyanobacteria*, genera *Subdoligranulum*, *Faecalibacterium*, *Ruminococcus*, and *Akkermansia* were decreased in CKD children stools with an abnormal ABPM profile.

CKD children with abnormal ABPM had a decreased proportion of genera *Gemella*, *Providencia*, and *Peptosreptoccocus*. Of note is that these genera of bacteria are involved in TMA production [92]. Accordingly, whether these microbes play a key role on the development of hypertension via the TMA−TMAO metabolic pathway in CKD children deserves further clarification.

In 20 children with INS who received oral prednisone therapy, abundance of genera *Romboutsia*, *Stomatobaculum*, and *Cloacibacillus* was increased after a 4-week initial therapy [87]. Another study recruited 20 children with INS and showed that probiotic treatment protected against relapse and coincided with increases in butyrate-producing bacteria and blood regulatory T cell (Treg) counts [89]. Considering gut microbiota shapes, the Th17/Treg balance, and Th17 involved in renal inflammation, probiotic treatment may have beneficial effects impacting the gut–kidney axis via immune regulation.

## 4. Gut Microbiota-Targeted Therapy

**Figure 2:**
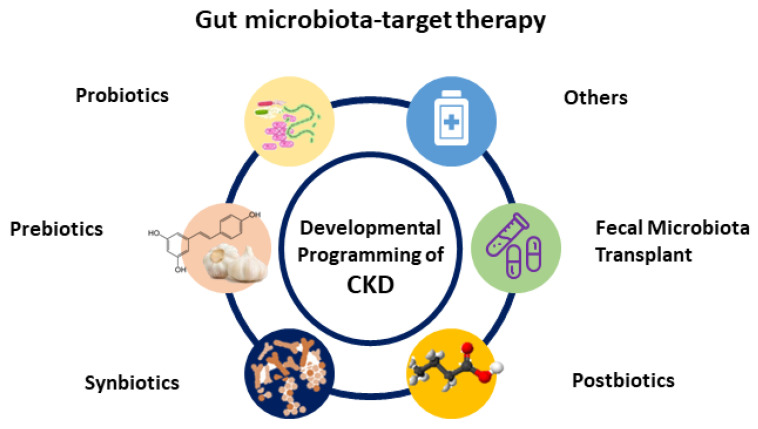
Schematic diagram of the potential gut microbiota-targeted therapy used for developmental programming of chronic kidney disease.

Recently, researchers have increasingly turned their attention on gut microbiota and its derived metabolites as a potential target for therapeutics [81,82,93,94]. In clinical practice, the most generally used gut microbiota-targeted therapies are probiotics and prebiotics. Probiotics are live bacteria that have health benefits when administered [93]. Prebiotics can promote the growth and activity of beneficial bacteria [93]. Synbiotics refer to a mixture comprising probiotic and prebiotics that also confers a health benefit. Additionally, the use of substances leased or produced through metabolism of the gut microbes, namely postbiotics, have shown a positive effect on the host [94]. Another gut microbiota-targeted therapy is fecal microbial transplantation (FMT). Although FMT is being broadly studied in microbiome-associated pathologies [95,96], its potential application for the treatment of CKD remains largely unknown. Moreover, treatment with oral intestinal absorbent AST-120 can reduce microbiota-derived uremic toxins [97]. Although AST-120 treatment has shown cardiovascular benefits in adult patients with CKD [98,99], its influence on gut microbiota compositions and other CKD-related complications remains limited. A summary of potential gut microbiota-targeted therapies in the treatment of developmental programming of CKD and its comorbidities is illustrated in Figure 2.

### 4.1. Human Evidence in Pediatric CKD

To date, limited data are available to examine whether alterations of gut microbiota by microbiota-targeted therapies can protect against CKD progression and its comorbidities in the pediatric population. For example, *Clostridium butyricum* is a butyrate-producing bacteria used as a probiotic [100]. Oral administration of *Clostridium butyricum* during remission was reported to reduce the frequency of relapse and the need for immunosuppressive agents in children with INS [89]. The protective effect of probiotic therapy was associated with increases in butyrate-producing bacteria and Treg cells. On the other hand, animal studies targeting gut microbiota to prevent the development of CKD and its associated complications have produced some compelling evidence. 

### 4.2. Animal Models of Early-Life Gut Microbiota-Targeted Therapy

Here, we list in Table 2 a summary of studies documenting gut microbiota-targeted interventions in animal models of CKD of developmental origins and its comorbidities [101,102,103,104,105,106,107,108,109,110]. The therapeutic duration is during fetal and childhood stages. The literature review states that gut microbiota-targeted interventions used to prevent CKD and its comorbidities primarily include probiotics, prebiotics, and postbiotics.

As shown in Table 2, rats are the dominant species used by experiments, and hypertension is the most commonly studied CKD-related comorbidity. A variety of early-life insults can lead to structural and functional changes in the developing kidney by the so-called renal programming [6]. Unlike in humans, kidney development in rats continues up to postnatal week 1–2. According to DOHaD theory, adverse environmental insults during pregnancy and lactation period can interrupt kidney development, resulting in renal programming and adult kidney disease. Several models of renal programming have been used to examine gut microbiota-targeted interventions in CKD of developmental origins, such as maternal high-fructose diet [101,108], perinatal high-fat diet [102,107,109], perinatal 2,3,7,8-tetrachlorodibenzo-p-dioxin (TCDD) exposure [103], maternal adenine-induced CKD [104], maternal TMAO and ADMA exposure [105], and maternal high-fructose diet and TCDD exposure [110].

Taking the example of the maternal high-fructose diet model, high-fructose intake during pregnancy and lactation modified over 200 renal transcripts from nephrogenesis stage to adulthood [111]. Using whole-genome RNA next-generation sequencing (NGS), high-fructose-induced alterations of the renal transcriptome were reported in kidneys from 1-day-, 3-week-, and 3-month-old male offspring. NGS identified genes in arachidonic acid metabolism (*Cyp2c23*, *Hpgds*, *Ptgds* and *Ptges*) that contribute to renal programming and hypertension. Notably, this renal programming model has been used to examine the reprogramming effects of gut microbiota-targeted therapy on fructose-induced developmental programming [112]. Since the above-mentioned renal programming models have been established and linked to adverse renal outcomes in adult offspring, readers are referred to original references. There was only one study conducting an adenine-induced pediatric CKD model to determine the effects of probiotic resveratrol on CKD progression [106]. 

Review elsewhere showed that several probiotic microorganisms and prebiotics have benefits on adult CKD [81,82], while there was only very limited evidence regarding their role on CKD of developmental origins. Supplementation with *Lactobacillus casei rhamnosus* from pregnancy through lactation protected adult male rat progeny against hypertension programmed by a maternal high-fructose diet [101] or perinatal high-fat diet [102].

Additionally, inulin as a prebiotic has been examined for its protective effect in hypertension of developmental origins [101,102]. In a high-fat model [102], we previously demonstrated that inulin treatment protected against hypertension in adult rat offspring coinciding with alterations of the gut microbiota, particularly increasing the abundance of *Lactobacillus*, a well-known probiotic strain. Likewise, perinatal supplementing to rat dams with inulin protected adult offspring against maternal high-fructose diet-induced hypertension, which coincided with an increased plasma level of propionic acid [102].

Resveratrol can modulate gut microbiota composition, undergo biotransformation to activate metabolites via the intestinal microbiota, affect gut barrier function, modify the *Firmicutes* to *Bacteroidetes* (F/B) ratio, and reverse the gut microbial dysbiosis [113,114,115,116]. With a prebiotic effect for gut microbes, increasing evidence supports the beneficial effects of resveratrol on many diseases, including CKD [117,118]. One study revealed that perinatal resveratrol therapy could protect adult offspring against hypertension and CKD of developmental origins [119]. Studies using a maternal TCDD exposure rat model showed TCDD-induced renal hypertrophy and hypertension in adult progeny, and both are key features of early CKD. TCDD-induced hypertension is associated with activation of AHR signaling, induction of TH17-dependent renal inflammation, and alterations of gut microbiota compositions [103]. Conversely, the induction of AHR- and TH17-mediated renal inflammation could be counterbalanced by perinatal resveratrol supplementation. The beneficial effects of resveratrol are associated with reshaping the gut microbiome by augmenting microbes that can inhibit TH17 responses and reduce the F/B ratio, a microbial marker of hypertension [14]. In a maternal CKD model, adult offspring developed renal hypertrophy and hypertension [104]. Perinatal resveratrol therapy protected hypertension, coinciding with the restoration of microbial richness and diversity and an increase in *Lactobacillus* and *Bifidobacterium* [104]. Similar to TMAO, asymmetric dimethylarginine (ADMA) is a well-known uremic toxin [120]. Another study using a maternal TMAO plus ADMA exposure model demonstrated that adult offspring born to dams exposed to uremic toxins had renal dysfunction and hypertension [105]. Conversely, maternal treatment with resveratrol rescued hypertension induced by TMAO plus ADMA exposure, accompanied by increased butyrate-producing microbes and fecal butyric acid level. 

Of note is that the low bioavailability of resveratrol diminishes its efficacy and clinical translation [121]. Accordingly, we produced resveratrol butyrate ester (RBE) via the esterification of resveratrol with the SCFA butyrate to improve the efficacy [122]. Using a pediatric CKD model [85], we recently found low-dose RBE (25 mg/L) is as effective as resveratrol (50 mg/L) in protecting against hypertension and renal dysfunction. The beneficial effects of RBE include regulation of SCFA receptors, decreased AHR signaling, and increased abundance of the beneficial microbes *Blautia* and *Enterococcus*.

Although there are many prebiotic foods, only garlic oil has shown beneficial effects against high-fat diet-induced hypertension in adult progeny [106]. These effects include increased α-diversity, increased plasma levels of acetic acid, butyric acid, and propionic acid, and increased beneficial bacteria *Lactobacillus* and *Bifidobacterium*.

In addition to probiotics and prebiotics, postbiotics is another gut microbiota-targeted therapy. Postbiotics include various components, such as microbial cell fractions, extracellular polysaccharides, functional proteins, cell lysates, extracellular vesicles, cell-wall-derived muropeptides, etc. [94]. Nevertheless, very limited information exists regarding the use of postbiotics in CKD. Acetate supplementation within gestation and lactation was reported to protect offspring against high-fructose-diet-induced hypertension, a major complication of CKD [108]. However, its protective effects on other complications of CKD are still waiting for clarification. Another example of postbiotic use in hypertension of developmental origins is conjugated linoleic acid [109]. Linoleic acid is a gut microbial metabolite derived from dietary polyunsaturated fatty acids (PUFA) [123]. Several gut microbes have been identified as producing PUFA-derived intermediate metabolites [124]. Administration of PUFA-derived bacterial metabolites such as linoleic acid has been shown to provoke anti-obesity and anti-inflammatory effects [125]. However, unlike probiotics and prebiotics [126,127], currently there is a lack of a clear definition for postbiotics. Considering the complex nature of postbiotics [94], a clear definition is important for future research from a regulatory perspective.

Moreover, there are other microbiota-related therapies applied for preventing CKD and its comorbidities. Microbe-dependent TMA and TMAO formation can be inhibited by 3,3-dimethyl-1-butanol (DMB), a structural analogue of choline [128]. Recently, two studies reported that maternal oral administration of DMB protected hypertension in adult rat progeny exposed to a maternal high-fructose diet [87] or high-fructose diet plus TCDD exposure [110]. This was accompanied by affecting the metabolic pathway of TMA-TMAO and reshaping gut microbiota [110].

As far as the multifaceted relationship between the gut and kidney, there might be other potential approaches by which the gut microbiota might prevent CKD and its associated complications. For example, RAS blockers are currently the most common therapies used for renoprotection and antihypertension [129]. Considering drug-mediated alterations in the gut microbiota compositions can have beneficial effects on the host [130], a greater understanding of mechanisms driving drug–gut microbiota interactions might aid in guiding the development of microbiota-targeted pharmacological interventions. Together, early microbiota-targeted therapies, in the long term, may enable the capacity to prevent the development of CKD and its comorbidities in a desired favorable direction. However, there is an urgent need to identify and fill the knowledge gaps on gut microbiota-targeted therapies between established CKD and CKD of developmental origins.

## 5. Conclusions and Perspectives

Mounting evidence in support of the link between gut microbiota and CKD starting in early life is intriguing but incomplete. One major unsolved problem is the gap in published child- and adult-focused clinical CKD research. Most pediatric CKD studies have limited power due to a small sample size. Although substantial evidence indicates an association between gut microbiota and CKD in adult patients with different stages of CKD and/or various comorbidities, we still lack such information in the pediatric population. Therefore, future work in large multicenter studies regarding CKD and its comorbidities is required to enable the establishment of more robust true relationships in the pediatric population.

Prior research has indicated that the early-life gut microbiome might influence renal programming and exert CKD in later life. Our review highlights the value of gut microbiota-targeted therapies, if applied early, to help prevent CKD and its related complications. Nevertheless, many probiotics and prebiotics used in adult CKD have not been examined in childhood CKD yet, especially in CKD of developmental origins.

In conclusion, gut microbiota dysbiosis is a highly pathogenetic link in the development of CKD and its comorbidities. After all of this significant growth in understanding of the gut microbiota in the pathophysiology of pediatric CKD and its targeted interventions, it may open new avenues for prevention of CKD in childhood or even earlier in fetal life.

## Figures and Tables

**Table 1:** Summary of studies investigated links between gut microbiota and pediatric chronic kidney disease.

Study	Study Population	Age (Years)	Alterations in Gut Microbiota and Metabolites
Crespo-Salgado et al., 2016 [83]	8 HD, 8 PD, 10 transplant, 13 controls	Control: 9.5 (3–16), HD: 13.6 (8–17), PD: 11.9 (3–17), transplant: 13.2 (2–18)	↓ Alpha diversity in PD and transplant ↓ Phyla *Firmicutes* and *Actinobacteria* but ↑ family *Enterobacteriaceae* in PD ↑ Phylum *Bacteroidetes* in HD ↑ Plasma levels of p-cresyl sulfate and indoxyl sulfate in HD and PD
Tsuji et al., 2018 [84]	12 INS, 11 controls	Controls: 5.1, relapsing INS: 3, non-relapsing INS: 4.3	↓ Butyrate-producing bacteria belonging to Clostridium clusters IV and XIVa ↓ Fecal butyric acid level
Hsu et al., 2018 [85]	60 CKD stage 1 26 CKD stage 2–3	11.3 (7.2–15.5) 11.3 (7.2–15.5)	↓ Urinary levels of DMA and TMAO in CKD stage 2–3 vs. CKD stage 1 ↓ Genus Prevotella in CKD children with an abnormal ABPM profile
Hsu et al., 2019 [86]	78 CKD stage 1–4	11.2 (7.4–15.2)	↑ Plasma levels of propionic acid and butyric acid in CKD children with an abnormal ABPM profile ↑ Phylum *Verrucomicrobia*, genus *Akkermansia*, and species ↓ *Bifidobacterium bifidum* in CKD children with CAKUT
Kang et al., 2019 [87]	20 INS	3.5 ± 2.1	↑ Genera *Romboutsia*, *Stomatobaculum* and *Cloacibacillus* after 4-week initial therapy
Hsu et al., 2020 [88]	115 CKD stage 1–4	11.3 (7.2–15.5)	↑ Plasma levels of DMA, TMA, and TMAO in children with CKD stage 2–4 vs. CKD stage 1 ↓ Phylum *Cyanobacteria*, genera *Subdoligranulum*, *Ruminococcus*, *Faecalibacterium*, and *Akkermansia* in CKD children with an abnormal ABPM profile
Yamaguchi et al., 2021 [89]	20 INS	INS with probiotics: 6.4 (3.7–10.6), INS without probiotics: 4.7 (3.5–7.8)	↓ Butyrate-producing bacteria

Data on age are presented as mean ± standard deviation or median (interquartile range); PD = peritoneal dialysis; HD = hemodialysis; CKD = chronic kidney disease; INS = idiopathic nephrotic syndrome; CAKUT = congenital anomalies of the kidney and urinary tract; DMA = dimethylamine; TMA = trimethylamine; TMAO = trimethylamine-N-oxide; ABPM = 24 h ambulatory blood pressure monitoring.

**Table 2:** Summary of early-life gut microbiota-targeted therapies for CKD and its comorbidities.

Gut Microbiota-Targeted Intervention	Animal Models	Species/Gender	Age at Evaluation	Effects on CKD and Its Comorbidities	Reference
Probiotics					
Daily oral gavage of *Lactobacillus casei rhamnosus* (2 × 10^8^ CFU/day) to mother rats from pregnancy through lactation	Maternal high-fructose diet	SD rat/M	12 weeks	Prevented hypertension	Hsu et al., 2018 [101]
Daily oral gavage of *Lactobacillus casei rhamnosus* (2 × 10^8^ CFU/day) to mother rats from pregnancy through lactation	Perinatal high-fat diet	SD rat/M	16 weeks	Prevented hypertension	Hsu et al., 2019 [102]
Prebiotics					
5% *w*/*w* long chain inulin to mother rats from pregnancy through lactation	Maternal high-fructose diet	SD rat/M	12 weeks	Prevented hypertension	Hsu et al., 2018 [101]
5% *w*/*w* long chain inulin to mother rats from pregnancy through lactation	Perinatal high-fat diet	SD rat/M	16 weeks	Prevented hypertension	Hsu et al., 2019 [102]
Resveratrol (50 mg/L) in drinking water to mother rats from pregnancy through lactation	Perinatal TCDD exposure model	SD rat/M	12 weeks	Prevented renal inflammation and hypertension	Hsu et al., 2021 [103]
Resveratrol (50 mg/L) in drinking water to mother rats from pregnancy through lactation	Maternal adenine-induced CKD	SD rat/M	12 weeks	Prevented hypertension	Hsu et al., 2020 [104]
Resveratrol (50 mg/L) in drinking water to mother rats from pregnancy through lactation	Maternal TMAO and ADMA exposure	SD rat/M	12 weeks	Prevented hypertension	Hsu et al., 2021 [105]
Resveratrol (50 mg/L) in drinking water to mother rats from week 6 to week 12	Pediatric adenine-induced CKD	SD rat/M	12 weeks	Prevented renal dysfunction and hypertension	Hsu et al., 2021 [106]
Resveratrol butyrate ester (25 mg/L or 50 mg/L) in drinking water to young rats from week 6 to week 12	Pediatric adenine-induced CKD	SD rat/M	12 weeks	Prevented renal dysfunction and hypertension	Hsu et al., 2021 [106]
Daily oral gavage of garlic oil (100 mg/kg/day) to mother rats from pregnancy through lactation	Perinatal high-fat diet	SD rat/M	16 weeks	Prevented hypertension	Hsu et al., 2021 [107]
Postbiotics					
Magnesium acetate (200 mmol/L) in drinking water to mother rats from pregnancy through lactation	Maternal high-fructose diet	SD rat/M	12 weeks	Prevented hypertension	Hsu et al., 2019 [108]
1% conjugated linoleic acid to mother rats from pregnancy through lactation	Maternal high-fat diet	SD rat/M	18 weeks	Prevented hypertension	Gray et al., 2015 [109]
Others					
1% DMB in drinking water to mother rats from pregnancy through lactation	Maternal high-fructose diet	SD rat/M	12 weeks	Prevented hypertension	Hsu et al., 2019 [108]
1% DMB in drinking water to mother rats from pregnancy through lactation	Maternal high-fructose diet and TCDD exposure	SD rat/M	12 weeks	Prevented hypertension	Hsu et al., 2020 [110]

Studies tabulated according to types of intervention, animal models, and age at evaluation. TCDD = 2,3,7,8-tetrachlorodibenzo-p-dioxin; CKD = chronic kidney disease; TMAO = trimethylamine-N-oxide; ADMA = asymmetric dimethylarginine; SD = Sprague-Dawley rat; DMB = 3,3-maternal dimethyl-1-butanol.

## Data Availability

All data are contained within the article.
